# Building Governmental Public Health Capacity to Advance Health Equity: Conclusions Based on an Environmental Scan of a Local Public Health System

**DOI:** 10.1089/heq.2019.0025

**Published:** 2020-08-24

**Authors:** Danielle L. Broussard, Maeve E. Wallace, Lisa Richardson, Katherine P. Theall

**Affiliations:** ^1^ Department of Global Community Health and Behavioral Sciences, Tulane University School of Public Health and Tropical Medicine, New Orleans, Louisiana, USA.; ^2^ Mary Amelia Douglas-Whited Community Women's Health Education Center, Tulane University School of Public Health and Tropical Medicine, New Orleans, Louisiana, USA.; ^3^ Institute of Women and Ethnic Studies, New Orleans, Louisiana, USA.

**Keywords:** community engagement, governmental public health, health equity, racial equity

## Abstract

Vast health inequities persist in cities across the United States. Although recommendations exist to guide governmental public health institutions seeking to advance population health equity, local contexts are likely to influence how these pursuits take shape. We review recommendations for pursuing equity that were developed from an environmental scan conducted in the city of New Orleans. The recommendations, which are based on perspectives provided by city and state public health leaders, leaders from other city governmental departments, and community-based health department partners, center around the enduring impact of systemic racism, working across sectors, and prioritizing community engagement.

## Introduction

Public health entities face numerous challenges to addressing health disparities from agendas driven by political pressure, to lack of funding aimed at the elimination of inequity. The persistence of health disparities underscores an urgent need to reconsider the effectiveness of traditional approaches to addressing the public's needs and explore broader policy options to achieve health equity.

In many cities across the country, the deep embedding of racial oppression is evident in the unequal distributions of a range of health-promoting resources and opportunities, neighborhood and social conditions, and generational accumulation of wealth.^1,2^ The result is a population health disadvantage among nonwhite and/or low-income groups that disproportionately lack access to resources to maintain health and to prevent disease.^3^

## The New Orleans Experience

We conducted an environmental scan of the New Orleans Health Department (NOHD) to identify practices and policies relevant to building the capacity of a local governmental public health agency to address health equity. The results of the scan, which were described in a 2019 report,^4^ were based on key informant interviews with NOHD staff, representatives from other city government departments, leadership within the Louisiana Department of Health, and community-based partners. In this study, we summarize key themes from those interviews and discuss our conclusions about barriers, needs, and opportunities identified at all societal levels,^5^ which can inform strategic efforts to advance health equity (Table 1).

**Table 1.** Summary of Barriers, Needs, and Opportunities Associated with Building the Capacity of Local Governmental Public Health to Address Health Equity Identified from Key Informant Interviews

Level of the socioecological model	Barriers	Needs	Opportunities
Systems/policy	Systems built on racism Social determinants drive health inequities Normalization of our history of racial inequity perpetuates inequity, which requires recognition and acknowledgement	Genuine political or governmental action to advance racial equity Prioritize community participation in systems change and initiatives	Partner with organizations that can lobby for change Partner with community members Use community organizing approaches to build support for health equity initiatives
Community	Lack of communication across government agencies	Share power more equitably with the community Coordinate communication across agencies/organizations doing similar work	Allow the community to lead efforts to end racial inequity Partner across sectors Find a common goal as a starting point for organizing/action
Organizations/institutions	Lack of communication with community Limited resources Assumptions about what community needs Workforce not prepared to address equity Extensive responsibilities of public health	Diversity in hiring within the health department Greater internal understanding of equity before engaging with the community Meaningful engagement to identify community needs Equity trainings as a continuous embedded processes	Equity in hiring practices and wages for health department staff Intentionality in diversifying the workforce Health department as a facilitator in ending inequity Partner with community members and groups in addition to community leaders Improve the quality of services provided
Interpersonal	Lack of understanding and empathy across groups	Adopt practices that demonstrate everyone has an equal voice	Ensure diversity and diversity of perspectives when convening groups to address health issues
Individual	Operating out of a mindset of denial about the existence of inequities	Training future leaders committed to creating a more equitable city	Support champions who are doing equity work

### Systems and policy level

A longstanding racial hierarchy that has shaped all aspects of society, including systems, and systemic racism and its impact on health were articulated in detail. For many informants, the first step toward achieving health equity was confronting the relationship between population-level health disparities and race. According to one informant,

There aren't any other ways of dividing populations—age, gender, that have as stark a disparity as race. We can't deal with any inequalities unless we are dealing primarily with racial inequalities.

There was, however, notable variation in opinion on the responsibility of the public health sector in pursuit of equity writ large. Several informants questioned the assumption that health equity should be the priority of governmental public health. As one informant commented,

….our primary focus is health. Equity is perhaps a byproduct of what we do.

Equity was never questioned as an ideal; however, it was noted that “the extensive responsibilities for governmental public health” and “limited resources including time, staff and funding” were the reasons that entities such as NOHD were often described as not being the primary lead, but rather as a facilitator in ending racial inequity.

### Community level

Effectively working in collaboration with community members was consistently described as a litmus test for the authenticity of governmental efforts. Although historically inequitable power dynamics and limited resources were again mentioned as obstacles, “meaningful community engagement” and deep engagement with residents was emphasized as an area that should receive more attention. One informant pointed out,

It has been said that we are good at engaging ‘grass tops but not grass roots.’ So, we know that we need to do a better job of engaging folks with the lived experience. But it takes much more time and effort.

Community engagement alone was not offered as the panacea for equity. In the words of another informant, “we need action that supports lip service.”

Emphasis was placed on the intersection of communication and community engagement. There were calls for both improved communication processes internal to NOHD as well as the need for better external communication to improve relationships, especially with communities of color, through sharing information about existing services, programs, and health promotion resources.

### Institutional level

Professional and personal commitments to interrogating organizational practices as essential to equity were clear, and leaders working to be intentional about changing organizational norms discussed practices that conflict with the goals of health equity. As one leader working in collaboration with NOHD noted,

There is often inverse relationship between racial equity and community engagement…some people consistently feel like they don't get to participate in plans, the development of solutions, or whatever the action steps are.

Officials steeped in the equity work of NOHD were more aware of the significant investments being made in improving hiring practices, ongoing professional development, and “norms-changing approaches.” For them, the increased capacity to address equity is evident:

We are thinking about community engagement differently, as in engaging the ‘internal community’ of the larger [governmental public health] organization. By engaging and changing the norms of providers and program staff, all of whom potentially reach many [community members], the impact can be tremendous.

It was noted that to be successful these efforts must be grounded in “in-depth training and a lot of internal discussions” about race, racism, and equity to “embed the orientation to equity” among staff.

### Interpersonal level

Trust was raised as a fundamental barrier to community engagement, effective communication, and representative diversity within public health. Trust frequently framed the discussion on systemic racism and the ability of public health agencies to facilitate a process to advance equity and work in true partnership with community to develop sustainable solutions to health challenges.

In addition, what appear to be intractable inequities were viewed as being rooted in a resistance to change that operates at the interpersonal level. One person said candidly,

I think people are concerned with what they would lose…it's almost as if this table was set and there has been another kiddie table over there, and now we are telling the people at the kiddie table they can come sit down. But the leadership at this current table has to figure out where they are going to fit.

A focus on commonalities and community strengths were offered as solutions to interpersonal challenges that were presented by multiple informants.

### Individual level

Respondents largely shared the view that critical to the success of equity work is the way people feel about equity as a societal goal. For those in leadership roles, the opportunity to affect change was magnified. As described by one informant,

The make-up of [public health] staff and leaders is so important—we need more diversity in the thinking and leadership roles…we need more strategic recruitment and mechanisms to enable the workforce to have the tough conversations about history.

Recruiting a public health workforce from varied backgrounds was emphasized as well as support for more equitable hiring practices and salaries. Connections between the themes of individual attitudes, orientation toward equity, and institutional practices that prioritize diversity were common. One public health leader stated,

……, if we want to serve this community, we should be reflective of this community. Not all black, and not all white, but we need to be black, white, Asian, and Hispanic. Everybody. Diversity is something you have to strive for and vigorously pursue.

## Conclusion

Three overarching themes emerged in our environmental scan, which served as the basis for our conclusions: (1) the long history and enduring impact of systemic racism, (2) the role of public health organizations and institutions in ending racial inequity, and (3) the importance of community relationships in the pursuit of equity.

Given broad acknowledgement that health systems and institutions were founded on racist frameworks, addressing racial inequities must precede the elimination of health inequities. Indeed, health outcomes are linked to numerous factors impacted by other sectors that shape and serve society, sectors that have also been influenced by discriminatory and oppressive principles.^6^ Increasing diversity and orienting the public health workforce toward an understanding of the legacy of racism in contributing to inequities has been recommended as a strategy to build organizational capacity to address equity.^7,8^

Governmental public health agencies in New Orleans and across the nation have engaged in cross-sector efforts to confront the factors that underlie health disparities, collaborations critical to advance health equity.^9–14^ The nature of these projects varies widely, but they are largely policy focused and include such efforts as targeting zoning policies to improve the built environment, conducting impact assessments to examine the potential community health impacts of policies, and advocating for citywide policies aiming to protect the public's health.

Finally, solutions for achieving equity must come from the community. Traditionally, decision-making about what communities need to be healthy has been dictated by the systems that serve them. These approaches have done little to build relationships with and garner trust from communities. Thus, community involvement in health equity initiatives may be enhanced by a paradigm shift in public health, from a siloed focus on disease prevention to one that centralizes social justice.^6,8^ These efforts should extend beyond engagement with community leaders and organizations and include community members who can offer the perspective of lived experience.^15^

Although our conclusions are based on a local example, they are consistent with strategies for advancing equity set forth by the Human Impact Partners and the Prevention Institute as well as those presented in the Department of Health and Human Services' Public Health 3.0.^8,9,13,16^ Our scan focused on the New Orleans context; however, similar assessments in other communities may promote local public health equity efforts.

## Abbreviation Used

NOHD: New Orleans Health Department
